# A re-randomisation design for clinical trials

**DOI:** 10.1186/s12874-015-0082-2

**Published:** 2015-11-05

**Authors:** Brennan C Kahan, Andrew B Forbes, Caroline J Doré, Tim P Morris

**Affiliations:** Pragmatic Clinical Trials Unit, Queen Mary University of London, London, E1 2AB UK; School of Public Health and Preventive Medicine, Monash University, Melbourne, VIC 3004 Australia; Comprehensive Clinical Trials Unit, University College London, London, WC1E 6BT UK; Hub for Trials Methodology Research, MRC Clinical Trials Unit at UCL, London, WC2B 6NH UK

**Keywords:** Clinical trial, Randomised controlled trial, Re-randomisation design, Re-enrolment, Poor recruitment

## Abstract

**Background:**

Recruitment to clinical trials is often problematic, with many trials failing to recruit to their target sample size. As a result, patient care may be based on suboptimal evidence from underpowered trials or non-randomised studies.

**Methods:**

For many conditions patients will require treatment on several occasions, for example, to treat symptoms of an underlying chronic condition (such as migraines, where treatment is required each time a new episode occurs), or until they achieve treatment success (such as fertility, where patients undergo treatment on multiple occasions until they become pregnant). We describe a re-randomisation design for these scenarios, which allows each patient to be independently randomised on multiple occasions. We discuss the circumstances in which this design can be used.

**Results:**

The re-randomisation design will give asymptotically unbiased estimates of treatment effect and correct type I error rates under the following conditions: (a) patients are only re-randomised after the follow-up period from their previous randomisation is complete; (b) randomisations for the same patient are performed independently; and (c) the treatment effect is constant across all randomisations. Provided the analysis accounts for correlation between observations from the same patient, this design will typically have higher power than a parallel group trial with an equivalent number of observations.

**Conclusions:**

If used appropriately, the re-randomisation design can increase the recruitment rate for clinical trials while still providing an unbiased estimate of treatment effect and correct type I error rates. In many situations, it can increase the power compared to a parallel group design with an equivalent number of observations.

**Electronic supplementary material:**

The online version of this article (doi:10.1186/s12874-015-0082-2) contains supplementary material, which is available to authorized users.

## Background

Patient recruitment is often a major challenge for randomised controlled trials (RCTs), and has been identified as the number one research priority by leads of UK trials units [1]. Reviews of publicly funded UK trials have found that between 45-69 % fail to successfully recruit to target [2, 3]. Another review of clinicaltrials.gov found that 48,027 patients had taken part in 481 trials that were at risk of being unable to address their primary research question due to poor recruitment [4].

Failure to recruit patients in a timely manner can have a major impact on patient care. It can lead to delays in completing trials, which in turn can cause delays in successful new treatments being adopted into routine clinical practice. It can increase trial costs, resulting in fewer trials being conducted. Finally, it can adversely impact the feasibility of conducting trials for conditions with a small patient pool. For example, a number of trials in patients with sickle cell disease have been terminated early due to insufficient recruitment [5]. As a result, care for patients with less common conditions may be based on suboptimal evidence from underpowered trials or non-randomised studies.

For many conditions, patients will require treatment on multiple occasions. For example, patients with an underlying condition for which symptoms recur will require treatment for each new presentation of symptoms (e.g. patients with sickle cell disease require pain relief each time they have a sickle cell pain crisis). Conversely, for some conditions an intervention may be given on a repeated basis until the patient is considered to be a treatment success (e.g. patients undergoing fertility treatments may undergo multiple treatment cycles before becoming pregnant).

The current norm for trials in these areas is for patients to be randomised once; they are ineligible to be re-enrolled into the trial a second time, even if they require further treatment. However, allowing each patient to be randomised on numerous occasions could increase the recruitment rate. This is called re-randomisation [6, 7], and has been used previously in trials of febrile neutropenia [8], irritable bowel syndrome [9], and influenza vaccines [10].

The properties of re-randomisation have been previously discussed for specific settings by Nason and Follman [11] and Dunning and Reeves [6]. This article describes the properties of the re-randomisation design, where each patient can be randomised multiple times, in a more general framework. We demonstrate that, under certain conditions, the re-randomisation design can provide unbiased estimates of treatment effect, correct type I error rates, and similar, or even increased power compared to that of a parallel group trial of the same size.

## Methods

### Overview of the re-randomisation design

The re-randomisation design is implemented as follows:

1. Patients are entered into the trial as usual, randomised to a treatment arm, and followed up until all primary and secondary outcomes have been collected
2. If a patient requires further treatment after completing their initial follow up period, and they still meet the inclusion/exclusion criteria, they may be entered into the trial again, and re-randomised
3. This is repeated until the target sample size is met

Instead of aiming to recruit a specified number of patients, the re-randomisation design aims to recruit a specified number of *observations*. Consider a required sample size of 200; instead of recruiting 200 patients, the re-randomisation design allows recruitment of fewer patients overall, provided the total number of times that these patients are entered into the trial is 200. For example, this could be achieved by randomising 100 patients once and 50 patients twice (200 observations from 150 patients), or by randomising 50 patients once and 50 patients 3 times (200 observations from 100 patients). A summary of the randomisation design is given in Table 1, and example data from a re-randomisation trial is given in Table 2.

**Table 1:** – Overview of the re-randomisation design

Implementing a re-randomisation design
1) Patients are entered into the trial as usual, randomised to a treatment arm, and followed up until all primary and secondary outcomes have been collected;
2) If a patient requires further treatment after completing their initial follow up period, they may be entered into the trial again, and re-randomised;
3) This is repeated until the target sample size is met.
Requirements for the re-randomisation design to give unbiased estimates of treatment effect and correct type I error rates
1) Patients are only re-randomised when they have completed the follow-up period from their previous randomisation;
2) Randomisations for the same patient are performed independently;
3) The treatment effect is constant across all randomisation periods.
Asymptotic properties of different analytical approaches
*Unadjusted analysis (ignoring patient effects)*
1) Unbiased estimate of treatment effect;
2) Correct type I error rate;
3) Equivalent power to a parallel group trial with the same number of observations in certain conditions (details provided in the text).
*Adjusted analysis (accounting for patient effects)*
1) Unbiased estimate of treatment effect (requires adjustment for number of previous allocations to both the intervention and control respectively when treatment effects carry over into subsequent randomisation periods);
2) Correct type I error rates;
3) Increased power compared to a parallel group trial with the same number of observations in most scenarios.

**Table 2:** – Example of patient data from re-randomisation trial

Patient ID	Randomisation period	Treatment allocation	Number of previous allocations to intervention (1)	Number of previous allocations to control (0)	Total number of times patient randomised
1	1	1	0	0	1
2	1	0	0	0	1
3	1	0	0	0	2
3	2	0	0	1	2
4	1	1	0	0	2
4	2	0	1	0	2
5	1	1	0	0	4
5	2	0	1	0	4
5	3	1	1	1	4
5	4	1	2	1	4
6	1	0	0	0	6
6	2	0	0	1	6
6	3	0	0	2	6
6	4	1	0	3	6
6	5	1	1	3	6
6	6	0	2	3	6

One key feature of the re-randomisation design is that the number of times each patient is randomised is not specified in advance. Instead, this will depend on the needs of each individual patient. For example, some patients may require treatment only once during the trial recruitment period; others several times. This means the re-randomisation design can be used in settings where the number of times a patient may require treatment is unknown. This is in contrast to a crossover trial, where every patient receives treatment a fixed number of times.

### Re-randomisation in the SWIM trial

We now discuss the concept of re-randomisation in the context of the SWIM (Sickle With Ibuprofen & Morphine) trial (ISRCTN97241637). SWIM compared the effectiveness of ibuprofen to placebo in reducing the amount of opioid consumed over four days for sickle cell patients who were receiving patient-controlled analgesia for an acute sickle cell pain crisis. Ibuprofen or placebo was given for a maximum of four days, and the follow-up period was for four weeks from hospital discharge. It was expected that most patients would be discharged within four days of randomisation, and so the follow-up period for most patients would be under five weeks. SWIM was designed as a parallel group trial, and patients could only be enrolled for one acute pain crisis. The target sample size was 316 patients. The SWIM trial had difficulties in patient recruitment and, as a result, was terminated early. Other trials in patients with sickle cell disease have faced similar issues in recruitment, and many have also terminated early [5].

Sickle cell disease is a lifelong condition, and many patients suffer from acute sickle cell pain crises on a recurring basis. Over a one-year period 46 patients presented 121 times with an acute pain crisis (number of presentations per patient ranged from 1–11) at one of the SWIM recruiting centres. 16 patients (35 %) presented only once, and 30 patients (65 %) presented on multiple occasions. The median number of presentations was 2 (IQR 1–3). Based on these numbers, a re-randomisation design for SWIM could have substantially increased the recruitment rate, increasing the feasibility of conducting such a trial.

### Properties of the re-randomisation design

We demonstrate in the following sections that the re-randomisation design will provide asymptotically unbiased estimates of treatment effect and correct type I error rates under the following conditions:

- Patients are only eligible for re-randomisation when the follow-up period from their previous randomisation is complete;
- Randomisations for the same patient are performed independently for each randomisation period;
- The treatment effect is constant across all randomisation periods.

Mathematically, these conditions can be expressed as follows.

Condition (a). Assume that the time of the *j*th randomisation is *t*_*j*_, and that the follow-up period for each randomisation period is *m*. Then, condition (a) requires that:

$$ {t}_j>{t}_{j-1}+m $$

Condition (b). Let *X*_*ij*_ be a random variable indicating which treatment the *i*th patient received during their *j*th randomisation period. Then, condition (b) requires:

$$ P\left({X}_{ij}=x\ \Big|\ {X}_{i,j-1}\right)=P\left({X}_{ij}=x\right),\kern0.75em x=0,1 $$

Condition (c). Let *β*_*j*_ be the treatment effect in randomisation period *j*. Then, condition (c) requires:

*β*_*j*_ = *β* for all *j.*

We discuss the rationale for each of these conditions below.

#### Patients are only eligible for re-randomisation when the follow-up period from their previous randomisation is complete

This condition is required to ensure there are no overlapping treatment periods where the same data could contribute to two separate observations. This is required for unbiased estimates of treatment effect.

For example, consider a trial with a one-year follow-up period. A patient is randomised to intervention, and then is re-randomised 3 months later to control; for the next 9 months, any data collected on this patient would contribute both to their initial randomisation to the intervention, and their subsequent randomisation to the control. The comparison between treatment and control is clearly invalidated in this scenario.

#### Randomisations for the same patient are performed independently

The probability of allocation to an arm must not depend on the patient’s allocations in their previous randomisation periods. This means that ‘patient’ should not be used as a stratification or balancing factor in the randomisation process (i.e. the randomisation procedure should not be ‘forced’ to assign each patient to the intervention and control an equal number of times). This is required for (a) unbiased estimates of treatment effect; and (b) correct type I error rates.

We first consider the issue of bias. Suppose the randomisation procedure was stratified by patient [12], so that those who received the control in their first randomisation period would receive the intervention in their next randomisation period, and vice versa. If only sicker patients who had received the control in the first randomisation period were re-randomised, all of these patients would receive the intervention in the second randomisation period. This would lead to a higher number of sicker patients in the intervention group, resulting in a biased comparison between treatment arms. Under independent randomisation however, the sicker patients who received the control in the first randomisation period and are then re-randomised would be equally likely to receive the intervention or control in the second randomisation period, ensuring comparable characteristics in both groups.

Secondly, we consider the issue of type I error rate. Observations from patients who are randomised multiple times will be correlated (that is, they will be more similar to each other than to observations from other patients). This can be viewed as a source of clustering (observations clustered within patients). Performing all randomisations for the same patient independently ensures that this clustering will be *ignorable*; that is, each observation can be treated as independent (despite the correlation) without adversely affecting the type I error rate (although this will lead to a reduction in power) [13]. Further details on why this is ignorable are available elsewhere [13].

In practice, randomisation can be performed by viewing each re-randomisation as a ‘new’ patient. A dummy randomisation sequence for a re-randomisation trial is shown in Table 2.

#### The treatment effect is constant across all randomisation periods

The treatment effect should be the same in each randomisation period. This means that a patient should receive the same benefit from the intervention (compared to control) each time they are randomised. This condition is required to ensure unbiased estimates of treatment effect.

When the treatment effect is not constant (i.e. the true treatment effect differs across randomisation periods), then a re-randomisation design will provide an ‘average’ treatment effect across periods. This could be misleading in some situations. For example, consider a trial testing an educational intervention. The entire benefit of this intervention is conferred the first time the patient receives it (as they are unlikely to learn anything new the 2^nd^ or 3^rd^ time they receive it). Therefore, an estimate of treatment effect which includes data from periods 2 and 3 will lead to substantial downward bias, and misleading results.

### Methods of analysis

There are two broad approaches to analysis which could be used with the re-randomisation design:

- An analysis which treats all observations as independent, even those from the same patient. We refer to this as an ‘unadjusted’ analysis, as it does not adjust for patient effects.
- An analysis which accounts for the correlation between observations from the same patient (for example, by including ‘patient’ as a random effect in a mixed-effects model). We refer to this as an ‘adjusted’ analysis, as it adjusts for patient effects.

We describe the properties of each of these analysis methods below, and a summary is given in Table 1. We focus here on continuous outcomes, but these results can easily be generalised to binary or time-to-event outcomes analysed using a logistic regression or Cox model.

#### Treating all observations as independent (unadjusted analysis)

In its general form, an unadjusted analysis for a continuous outcome can be written as:

1 $$ {Y}_{ij}=\alpha +\beta {X}_{ij}+{\varepsilon}_{ij} $$

where *Y*_*ij*_ is the outcome from the *j*^th^ randomisation period for the *i*^th^ patient, *X*_*ij*_ is a binary variable indicating which treatment was received, *β* is the treatment effect, and *ε*_*ij*_ is a random error term which follows a normal distribution with mean 0. This could be implemented using a linear regression model.

The unadjusted analysis will have the following asymptotic properties, provided the conditions from the previous section are fulfilled:

- Unbiased estimate of the treatment effect
- Correct type I error rate
- Equivalent power to a parallel group trial with the same number of observations, under certain extra conditions (discussed below)

We discuss each of these properties below.

#### Treatment effect estimate

The treatment effect from an unadjusted analysis, $\hat{\beta}$, can be calculated by subtracting the mean of the outcomes in one group from the mean in the other group. Because the treatment assignment is independent of patient, any systematic differences in baseline characteristics between randomisation periods, or between patients who are re-randomised and those who are not, will average out between treatment groups. Therefore:

$$ E\left(\hat{\beta}\right)=\beta $$

This property will hold even if there are systematic differences across randomisation periods, between patients who are re-randomised and those who are not, or if the treatment effect carries over into subsequent randomisation periods. We demonstrate this for the case when patients can be randomised up to two times in the Additional file 1.

#### Type i error rate

The type I error rate will be asymptotically correct. This is because, as discussed earlier, the clustering of observations within patients is a form of *ignorable* clustering, and therefore does not need to be accounted for in the analysis to obtain valid error rates. Further details on this are provided elsewhere [13].

#### Power

Under certain conditions, an unadjusted analysis will have equivalent power to a parallel group design (analysed using model (1)) with the same number of observations. This is because the variance of both treatment effect estimates are the same. Formally:

$$ \mathrm{V}\mathrm{a}\mathrm{r}\left({\hat{\beta}}_{RR}\right)=\mathrm{V}\mathrm{a}\mathrm{r}\left({\hat{\beta}}_{PG}\right) $$

where ${\hat{\beta}}_{RR}$ and ${\hat{\beta}}_{PG}$ are the treatment effect estimates from a re-randomisation and a parallel group design respectively. This occurs when patient outcomes (*Y*_*ij*_) take the following form:

2 $$ {Y}_{ij}=\alpha +\beta {X}_{ij}+{u}_i+{\varepsilon}_{ij} $$

where *u*_*i*_ is a random-effect for the *i*th patient (generally assumed to follow a normal distribution with mean 0), and the probability of being re-enrolled does not depend on the allocation or observed outcome from previous randomisation periods, or on the patient-specific random-effect (*u*_*i*_).

A proof of this for the case where patients are enrolled up to two times is given in the Additional file 1, but the essence is that the loss in precision from patients re-randomised to the same treatment arm is offset by the gain in precision from patients re-randomised to the opposing treatment arm.

Some of the key conditions implied by model (2) that allow the power from an unadjusted analysis to be the same as that from a parallel group trial are:

I. Patients’ expected outcomes are the same across randomisation periods, conditional on any covariates included in the analysis
II. The within-patient variation is the same across randomisation periods
III. The probability of being re-enrolled does not depend on the treatment allocation or observed outcome from previous randomisation periods, or on the patient’s underlying health status

Mathematically, these conditions can be expressed as follows.

Condition (i) Let *Z*_*ij*_ represent a set of covariates that are included in the analysis model, and *j* ≠ *j*^'^ represent different randomisation periods. Then:

$$ E\left({Y}_{ij}\ \Big|\ {X}_{ij}=x,{Z}_{ij}=z\right) = E\left({Y}_{i{j}^{\hbox{'}}}\ \Big|\ {X}_{i{j}^{\hbox{'}}}=x, {Z}_{i{j}^{\hbox{'}}}=z\ \right) $$

Condition (ii) This requires:

*V*(*ε*_*ij*_) = *σ*_*ε*_^2^ for all *j.*

Condition (iii) Let *R*_*ij*_ indicate whether the *i*th patient was enrolled into the trial during the *j*th randomisation period (where 1 denotes the patient was re-enrolled, and 0 indicates they were not), and *c* denote the current randomisation period. Then:

$$ P\left({R}_{ic}=1\ \Big|\ {u}_i,{Y}_{ij},{X}_{ij}\right) = P\left({R}_{ic}=1\right)\kern1.25em for\  all\ j<c $$

If these conditions do not hold (or when patient outcomes do not take the form of model (2)), then a re-randomisation design with an unadjusted analysis will generally lose power compared to a parallel group trial with the same number of observations. The amount of power lost will depend on how extreme the violation of these conditions is. However, in some cases power can be recovered by adjusting for any covariates which may explain these differences. For example, if outcomes are expected to differ by randomisation period, including randomisation period in the model will increase power [14–16].

#### Accounting for patient effects (adjusted analysis)

There are several different methods of analysis which can account for the correlation between observations from the same patient. The most common are generalised estimating equations (GEE) [17] and mixed-effects models [18]. For simplicity, we focus here on mixed-effects models, although for continuous outcomes we expect results for GEEs to be similar, as the treatment effect estimates for both models will coincide.

A mixed-effects model with a continuous outcome takes the same general form as model (2), i.e:

$$ {Y}_{ij}=\alpha +\beta {X}_{ij}+{u}_i+{\varepsilon}_{ij} $$

where *u*_*i*_ is a random-effect for the *i*th patient, and is generally assumed to follow a normal distribution with mean 0. The intraclass correlation (ICC), representing the correlation between repeated measurements on each subject, is defined as $\frac{\mathrm{Var}\left({u}_i\right)}{\mathrm{Var}\left({u}_i\right)+\mathrm{V}\mathrm{a}\mathrm{r}\left({\epsilon}_{ij}\right)}$. This can easily be generalised to a mixed-effects logistic regression model for binary outcomes or a frailty model for time-to-event outcomes.

The adjusted analysis will have the following asymptotic properties, provided the conditions from the earlier ‘*Properties of the re-randomisation design’* section are fulfilled (no overlapping follow-up periods, independent randomisation, and constant treatment effect across randomisation periods):

- Unbiased estimate of treatment effect (although adjustment for the number of previous allocations to intervention and control is required in certain cases; more details are provided below)
- Correct type I error rates
- Increased power compared to parallel group trials under certain conditions.

We discuss each of these properties below.

#### Treatment effect estimate

For a mixed-effects model, the overall treatment effect is calculated by combining the within-patient and between-patient estimates (weighted by the inverse of their variances) [18]. The overall treatment effect will therefore be unbiased if these components are unbiased. If any component is biased, than the overall treatment effect will also likely be biased, although not necessarily to the same extent.

We show in the Additional file 1 that an adjusted analysis will lead to unbiased estimates of treatment effect in most scenarios, including situations when:

- There are differences in outcomes across randomisation periods;
- There are differences in outcomes between single and multiple randomised patients;
- Only patients who received the intervention (or only patients who received the control) are re-randomised;
- Only patients who experienced a poor outcome in their initial randomisation period are re-randomised.

However, when the effects of different treatment arms differentially carry over into subsequent randomisation periods (e.g. when treatments change patients’ expected outcomes for future randomisation periods in different ways), the analysis must account for the number of previous allocations to each treatment arm to obtain unbiased estimates of treatment effect (these covariates are represented in columns 4 and 5 in Table 2).

We define carry over as follows. Let *c* denote the current randomisation period, and *f(y)* as a distribution function for *y*. Then:

$$ f\left({Y}_{ic}\ \Big|\ {X}_{ic},\ {X}_{ij}\right)\ne f\left({Y}_{ic}\Big|{X}_{ic}\right)\kern1.25em for\  all\ j<c $$

i.e. the distribution of the patient’s outcome in their current randomisation period *c* depends on which treatment they were allocated to in their previous randomisation periods.

Carry over could occur if the intervention (but not the control) permanently improves patients’ health, so patients who received the intervention during the first randomisation period will have better outcomes in the second randomisation period than patients with the same allocation in the second period but who received the control during the first randomisation period. Other scenarios are also possible. For example, both the intervention and control could permanently improve patient’s health, but could do so by different amounts. Alternatively, they may improve patient’s health by similar amounts, but the effects of the intervention may be longer lasting than the control.

#### Type i error rate

This analysis method should asymptotically provide correct type I error rates for all scenarios in which the estimated treatment effect is unbiased. However, it has been previously noted that GEEs and mixed-effects models have led to increased error rates in small sample situations [19, 20]. Therefore, with a small number of patients, a small-sample correction should be used.

#### Power

**Fig. 1:**
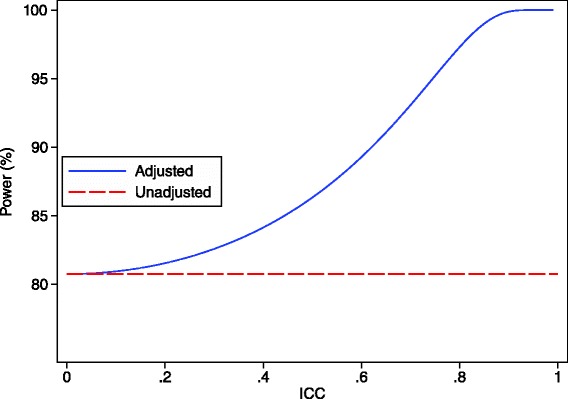
Analytical results showing the increase in power through a re-randomisation design for different ICC values. This graph shows the difference in power between an unadjusted analysis (ignoring patient-effects) and an adjusted analysis (accounting for patient-effects) for a re-randomisation design across different ICC values. There are 100 patients who are randomised once, and 50 patients who were randomised twice (200 overall observations), and the treatment effect is 0.40, with the within-patient standard deviation set to 1

**Fig. 2:**
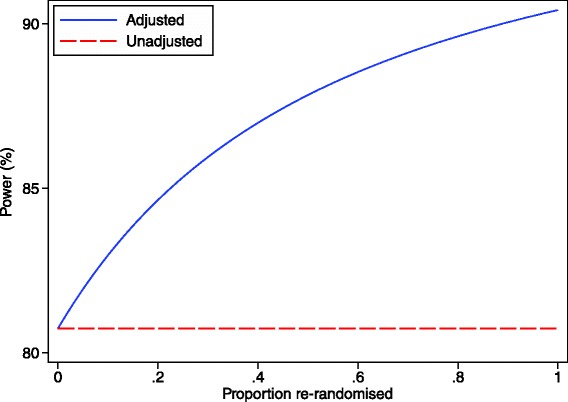
Analytical results showing the increase in power through a re-randomisation design for different re-randomisation rates. This graph shows the difference in power between an unadjusted analysis (ignoring patient-effects) and an adjusted analysis (accounting for patient-effects) for a re-randomisation design across different re-randomisation rates. The number of total observations is fixed at 200; the number of individual patients is calculated as: total observations/(1 + proportion of patients re-randomised). The ICC is 0.50, the treatment effect is 0.40, with the within-patient standard deviation set to 1

Because observations from the same patient are likely to be correlated in most scenarios, adjusting for patient effects in the analysis can lead to increased power compared to a parallel group trial with the same number of observations [16]. This will occur when the weight given to the within-subject treatment comparisons is greater than in the unadjusted analysis (which we have shown has the same variance as a parallel group trial of the same size). The extent to which power can be increased depends on the number of observations per patient and the within-patient correlation. This is shown in Figs. 1 and 2. One scenario where an adjusted analysis may not provide a large increase in power is when there are very few patients who have been re-randomised.

### Simulation study

We performed a simulation study to evaluate the performance of the re-randomisation design in a variety of scenarios. We assessed the impact of different intraclass correlation coefficients (ICC) and different re-randomisation rates. We also assessed a number of scenarios where a non-random subset of patients was re-randomised, or when treatment effects differentially carried over into subsequent randomisation periods.

We generated continuous outcomes from model (2) above:

$$ {Y}_{ij}=\alpha +\beta {X}_{ij}+{u}_i+{\varepsilon}_{ij} $$

where *Y*_*ij*_ is the outcome for the *j*th randomisation period from the *i*th patient, *β* is the effect of treatment, *X*_*ij*_ is a binary variable indicating whether the patient received the treatment or control, *u*_*i*_ is the random effect for the *i*th patient, and *ε*_*ij*_ is an error term for the *j*th observation from the *i*th patient. Both *u*_*i*_ and *ε*_*ij*_ follow normal distributions with mean 0 and variance *σ*_*u*_^2^ and *σ*_*ε*_^2^ respectively, and were generated independently. For all simulation scenarios we fixed the total variance as *σ*_*u*_^2^ + *σ*_*ε*_^2^ = 1. We used simple randomisation to allocate observations to either the intervention or control arm.

For each simulation scenario we compared two methods of analysis; (a) ignoring patient effects (unadjusted analysis, using model (1)), and (b) accounting for patient effects using a mixed-effects regression model, with patient included as a random-effect (adjusted analysis, using model (2)). For the adjusted analysis based on a mixed-effects model, we calculated p-values using a *t*-distribution, with degrees of freedom calculated as the total number of patients and fixed parameters in the model subtracted from the total number of observations [21].

For each method of analysis, we evaluated the estimated treatment effect, the type I error rate, and the power. We set the treatment effect to give 80 % power for a parallel group trial with no re-randomisation based on the specific simulation scenario. We used 5000 replications for each scenario, in order to provide standard errors of 0.3 % and 0.6 % for the estimated type I error rate and power respectively (assuming true values of 5 % and 80 %). All simulations were performed using Stata 13.1. We repeated the first two scenarios (varying the ICC and varying the proportion of re-randomisations) with a binary outcome – full details of the methods and results are presented in the Additional file 1.

#### Varying the ICC

We generated outcomes from model (2). We varied the ICC between 0.10, 0.25, 0.50, 0.75, and 0.90, while keeping the total variance (*σ*_*u*_^2^ + *σ*_*ε*_^2^) fixed at 1 (i.e. for increasing ICCs, we increased *σ*_*u*_^2^ while reducing *σ*_*ε*_^2^). For each scenario, we kept the proportion of re-randomisations fixed. We used 200 observations (100 patients randomised once, 50 patients randomised twice). For each ICC value we also evaluated a set of completely independent observations (200 patients randomised once) to compare the re-randomisation and parallel group designs.

#### Varying the proportion of re-randomisations

We generated outcomes from model (2). We varied the proportion of randomisations as follows: (a) 100 patients randomised once, 50 patients randomised twice; (b) 100 patients randomised once, 25 patients randomised four times; (c) 100 patients randomised twice; (d) 50 patients randomised four times; and (e) 25 patients randomised eight times. For each scenario we set the ICC to 0.50.

#### Scenarios where a non-random subset is re-randomised, or outcomes differ across randomisation periods

We assessed a number of scenarios where the patients who are re-randomised are systematically different than those who are not, or when patients are in different health states for each randomisation. We set the ICC to 0.50 for all scenarios.

We evaluated seven different scenarios:

- Scenario 1: Patients who are re-randomised are sicker than those who are not
- Scenario 2: Patients who experienced a poor outcome during their first randomisation are more likely to be re-randomised
- Scenario 3: Patients who received the intervention during their first randomisation are more likely to be re-randomised
- Scenario 4: Patients who received the control during their first randomisation are more likely to be re-randomised
- Scenario 5: Patients’ health status changes for their subsequent re-randomisation period
- Scenario 6: The intervention effect carries over into subsequent randomisation periods
- Scenario 7: The intervention and control effects carry over into subsequent randomisation periods by different amounts

Scenario 1: Sicker patients re-randomised We used 200 observations (100 patients randomised once, 25 patients randomised four times). We generated outcomes from the following model:

$$ {Y}_{ij}=\alpha +\beta {X}_{ij}+\theta {Z}_{ij}+{u}_i+{\varepsilon}_{ij} $$

where *Z*_*ij*_ is a binary variable indicating whether the patient requires re-randomisation or not, and *θ* indicates the average difference in outcomes between patients who were re-randomised compared to those who were not. We set *θ* = − 0.5.

Scenario 2: Patients who experience a poor outcome are re-randomised We generated 150 independent patients using model (2). Patients with *Y*_*ij*_ < −0.44 were re-randomised one more time. We selected −0.44 as a cut-off as this corresponded to approximately 33 % of patients being re-randomised (leading to an average of 200 observations).

Scenario 3: Intervention patients more likely to be re-randomised We generated 130 independent patients using model (2). All patients who received the intervention were re-randomised once more; patients who received the control were not re-randomised. This led to an average of 195 observations.

Scenario 4: Control patients more likely to be re-randomised This was performed the same way as above, except control patients were re-randomised and intervention patients were not.

Scenario 5: Period effect (patients’ health status changes for subsequent re-randomisation periods) We used 200 observations (50 patients randomised four times each). We generated outcomes from the following model:

$$ {Y}_{ij}=\alpha +\beta {X}_{ij}+\pi {Z}_{ij}+{u}_i+{\varepsilon}_{ij} $$

where *Z*_*ij*_ is a categorical variable indicating the re-randomisation period (0, 1, 2, or 3), and *π* represents the change in outcome for that re-randomisation period. We set *π* =0.5. During the analysis, we adjusted for re-randomisation period as an indicator variable.

Scenario 6: The intervention effect carries over into subsequent randomisation periods We used 200 observations (50 patients randomised four times each). We generated outcomes from the following model:

$$ {Y}_{ij}=\alpha +\beta {X}_{ij}+\theta {A}_{ij}+{u}_i+{\varepsilon}_{ij} $$

where *A*_*ij*_ is a variable indicating the number of times the patient has been allocated to the intervention in their previous randomisations (from 0–3; this variable is represented in column 4 of Table 2), and *θ* is the effect on outcome for each additional previous time allocated to the intervention. We set *θ* =0.4 (equivalent to the treatment effect). Formally:

$$ {A}_{ij}={\displaystyle \sum_{j=1}^{c-1}}I\left({X}_{ij}\right) $$

where *c* denotes the current randomisation period, and *I*(*X*_*ij*_) = 1 if *X*_*ij*_ = 1 and *I*(*X*_*ij*_) = 0 if *X*_*ij*_ = 0.

In this scenario, the intervention increases the patient’s outcome by 0.4, and this benefit carries through into subsequent randomisation periods. Conversely, the control has no effect on outcome.

We performed three analyses for this scenario: (a) an unadjusted analysis using model (1) that did not adjust for *A*_*ij*_; (b) an adjusted analysis using model (2) that did not adjust for *A*_*ij*_; and (c) an adjusted analysis using model (2) that adjusted for *A*_*ij*_ as an indicator variable.

Scenario 7: The intervention and control effects carry over into subsequent randomisation periods by different amounts We used 200 observations (50 patients randomised four times each). We generated outcomes from the following model:

$$ {Y}_{ij}=\alpha +\beta {X}_{ij}+\theta {A}_{ij}+\lambda {B}_{ij}+{u}_i+{\varepsilon}_{ij} $$

Where *A*_*ij*_ and *B*_*ij*_ represent the number of times the patient has been allocated to the intervention and control respectively in their previous randomisation periods (from 0–3; these variables are represented in columns 4 and 5 of Table 2), and *θ* and *λ* represent the effects on outcome for each additional previous time allocated to the intervention and control respectively. We set the overall treatment effect to *β* = 0.4, as in previous scenarios. We set *θ* =0.6 and *λ* =0.2, representing the change from baseline for each previous allocation to intervention and control. Formally:

$$ {A}_{ij}={\displaystyle \sum_{j=1}^{c-1}}I\left({X}_{ij}\right)\kern0.75em \mathrm{and}\kern1em {B}_{ij}={\displaystyle \sum_{j=1}^{c-1}}I\left({X}_{ij}\right) $$

We performed four analyses for this scenario: (a) an unadjusted analysis using model (1) that did not adjust for either *A*_*ij*_ or *B*_*ij*_; (b) an adjusted analysis using model (2) that did not adjust for either *A*_*ij*_ or *B*_*ij*_; (c) an adjusted analysis using model (2) that adjusted for *A*_*ij*_ as an indicator variable, but did not adjust for *B*_*ij*_; and (d) an adjusted analysis using model (2) that adjusted for both *A*_*ij*_ and *B*_*ij*_ using indicator variables.

#### A small number of patients

We performed a set of simulations to assess the performance of re-randomisation with a small number of patients. We generated outcomes from model (2). We varied the number of patients between 5, 10, 15, 20, 25, and 30, and the number of randomisation periods for each patient between 2, 4, and 8. We assessed both simple randomisation, and permuted blocks stratified by randomisation period. We performed two methods of analysis (both unadjusted for patient-effects, based on model (1)); the first ignored randomisation period, and the second adjusted for it using indicator variables. For each scenario we set the ICC to 0.75. We set the treatment effect to 0 for all scenarios to assess the type I error rate.

## Results

### Simulation results

#### Varying the ICC and the proportion of re-randomisations

**Fig. 3:**
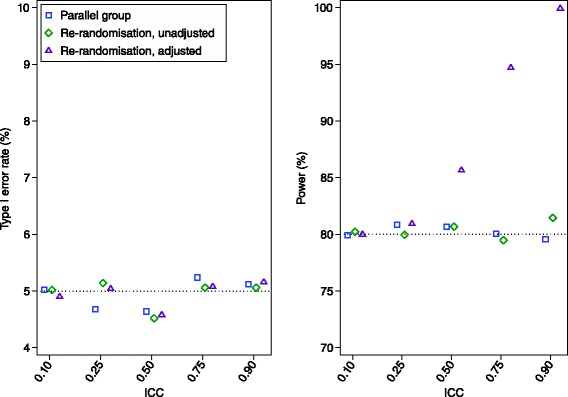
Simulation results across different ICC values. We compared three methods of analysis: (a) analysis of a parallel group trial with 200 independent patients; (b) an unadjusted analysis (ignoring patient effects) of a re-randomisation design, with 100 patients randomised once, and 50 patients randomised twice; and (c) an adjusted analysis (accounting for patient effects using a mixed-effects model) of a re-randomisation design, with 100 patients randomised once, and 50 patients randomised twice. The treatment effect estimates from all three methods of analysis were unbiased. Standard errors for the estimated type I error rate and power are 0.3 % and 0.6 % respectively (assuming true values of 5 % and 80 %)

**Fig. 4:**
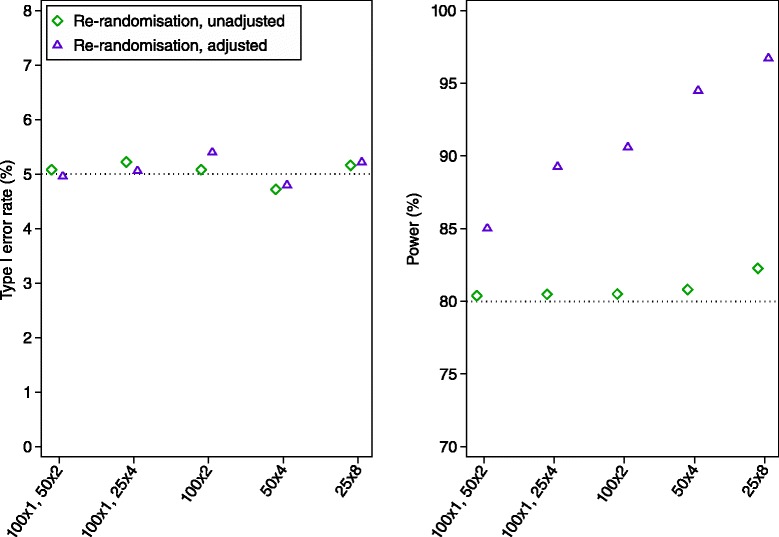
Simulation results across different re-randomisation proportions We compared two methods of analysis: (a) an unadjusted analysis (ignoring patient effects); and (b) an adjusted analysis (accounting for patient effects using a mixed-effects model). The ICC was set to 0.50 for all scenarios. The estimated treatment effect was unbiased for both methods of analysis. Standard errors for the estimated type I error rate and power are 0.3 % and 0.6 % respectively (assuming true values of 5 % and 80 %)

Results are shown in Figs. 3 and 4. An analysis which treated all observations as independent (unadjusted analysis) led to unbiased estimates of treatment effect, correct type I error rates, and identical power to a parallel group design in all scenarios.

Accounting for patient-effects using a mixed-effects model (adjusted analysis) also led to unbiased estimates of treatment effect and correct type I error rate in all scenarios. In most scenarios, it led to higher power than a parallel group trial (>10 % absolute increase in some cases). Gains in power were most pronounced when there was a high ICC, or a large proportion of re-randomisations.

Simulation results for binary outcomes are shown in the Additional file 1. Results were similar to simulations involving continuous outcomes.

#### Scenarios where a non-random subset is re-randomised, or outcomes differ across randomisation periods

Results are shown in Table 3. An unadjusted analysis led to unbiased estimates and correct type I error rates in all scenarios. The loss in power compared to a parallel group trial was 1.2 % or less in 5 of 7 scenarios, and <3.0 % in 6 of 7 scenarios. However, when the intervention and control effects carried over differentially into subsequent randomisation periods (scenario 7), an unadjusted analysis lost 10.3 % power compared to a parallel group design.

**Table 3:** – Simulation results for scenarios where a non-random subset is re-randomised, outcomes differ across randomisation periods, or treatment effects carryover into subsequent randomisation periods^a^

	Treatment effect = 0		Treatment effect = 0.4	
	Estimated treatment effect	Type I error rate (%)	Estimated treatment effect	Difference in power vs. parallel group trial^b^ (%)
Scenario 1: Sicker patients are re-randomised				
• Unadjusted for patient effects	0.0	5.1	0.4	−0.4
• Adjusted for patient effects^c^	0.0	5.2	0.4	+10.8
Scenario 2: Patients who experienced a poor outcome are more likely to be re-randomised				
• Unadjusted for patient effects	0.0	4.4	0.4	−1.2
• Adjusted for patient effects^c^	0.0	4.8	0.4	+6.1
Scenario 3: Patients who received the intervention are more likely to be re-randomised				
• Unadjusted for patient effects	0.0	5.0	0.4	−0.3
• Adjusted for patient effects^c^	0.0	4.8	0.4	+7.3
Scenario 4: Patients who received the control are more likely to be re-randomised				
• Unadjusted for patient effects	0.0	4.7	0.4	−0.3
• Adjusted for patient effects^c^	0.0	5.4	0.4	+7.0
Scenario 5: Patients’ health status changes for their subsequent re-randomisation^d^				
• Unadjusted for patient effects	0.0	5.3	0.4	−1.0
• Adjusted for patient effects^c^	0.0	5.1	0.4	+13.9
Scenario 6: The intervention effect carries over into subsequent randomisation periods				
• Unadjusted for patient effects	NA	NA	0.4	−2.9
• Adjusted for patient effects^c^	NA	NA	*0.3*	*−22.9*
• Adjusted for patient effects^c^, and adjusted for number of previous allocations to the intervention	NA	NA	0.4	+13.3
Scenario 7: The intervention and control effects carry over differentially into subsequent randomisation periods				
• Unadjusted for patient effects	NA	NA	0.4	−10.3
• Adjusted for patient effects^c^	NA	NA	*0.3*	*−29.8*
• Adjusted for patient effects^c^, and adjusted for number of previous allocations to the intervention	NA	NA	*0.5*	*+18.4*
• Adjusted for patient effects^c^, and adjusted for both the number of previous allocations to the intervention and number of previous allocations to the control	NA	NA	0.4	+10.0

^a^The ICC was set to 0.50 for all scenarios. The number of observations was 200 for scenario 1 (100 patients randomised once, 25 patients randomised 4 times), 200 on average for scenario 2 (approximately 100 patients randomised once, 50 randomised twice), 195 on average for scenarios 3 and 4 (approximately 65 patients randomised once, 65 patients randomised twice), and 200 for scenarios 5, 6, and 7 (50 patients randomised four times)

^b^Power for the parallel group trial was set at 80 %

^c^Analyses adjusted for patient-effects using a mixed-effects linear regression model, with a random intercept for patient

^d^Both analyses adjusted for randomisation period as an indicator variable

Adjusting for patient-effect using a mixed-effects model led to unbiased estimates and correct type I error rates for scenarios 1–5. In these scenarios, it led to greatly increased power compared with a parallel group trial (range 6.1–13.9 %). In scenarios 6 and 7 (when the intervention and/or control effects carried over into subsequent randomisation periods), the mixed-effects model required adjustment for the number of previous allocations to intervention and/or control in order to obtain unbiased estimates of treatment effect. This analysis strategy still lead to increased power compared to a parallel group trial (13.3 % and 10.0 % increases for scenarios 6 and 7 respectively).

#### A small number of patients

Full results are shown in the Additional file 1. An unadjusted analysis led to an increased type I error rate when there was a very small number of patients and randomisation was performed using permuted blocks stratified by randomisation period. However, type I error rates were correct when simple randomisation was used, when there were approximately 15 or more patients, or when randomisation period was included as a covariate in the analysis model.

### Further issues to consider with re-randomisation

We now discuss some further issues to consider when planning a trial using a re-randomisation design. These issues include: (a) when can the re-randomisation design be used in practice; (b) which covariates we should adjust for in the analysis of a trial utilising re-randomisation; (c) how to choose between a re-randomisation and crossover design; (d) potential issues when using re-randomisation in an open-label trial; (e) the number of times a patient can be re-randomised; (f) after what length of time a patient can be re-randomised; (g) how to consent patients; and (h) what to do when patients are accidentally re-randomised in a parallel group trial.

### When can the re-randomisation design be used in practice?

There are two key factors which will determine whether the re-randomisation design can be used in practice:

- The length of the follow-up period in relation to the overall length of the recruitment period.
- Whether the assumption of constant treatment effect across randomisation periods is likely to hold.

We discuss each of these issues in turn.

#### The length of the follow-up period

In order for re-randomisation to be beneficial, the follow-up period for each patient must be short in relation to the overall recruitment period. Consider a trial where the follow-up period is one month, and the overall recruitment period is expected to last for two years. Patients recruited at the beginning of the trial could be enrolled numerous times; similarly, patients recruited towards the end of the recruitment period could also be enrolled several times. Depending on how often patients require treatment, re-randomisation has the potential to substantially increase the recruitment rate in this scenario.

Conversely, consider a trial with a follow-up period of 18 months and recruitment period of two years. Only patients recruited during the first six months of the recruitment period could be enrolled a second time. In this scenario, relatively few patients are likely to be re-randomised, and so a re-randomisation design is unlikely to be beneficial.

#### The assumption of a constant treatment effect across randomisation periods

The key assumption required for the re-randomisation design to provide an unbiased estimate of treatment effect is that the treatment effect is constant across randomisation periods. Therefore, we suggest the re-randomisation design only be used in situations where there is reason to believe this assumption will hold. This indicates that a number of intervention types will be inappropriate to use in a re-randomisation design. Examples include interventions which can only be performed once (e.g. a type of surgery that permanently changes a part of the body) or interventions where the entire benefit is conferred during the first treatment period (e.g. an educational intervention that is learnt during the first allocation, and cannot be learned again).

However, there may be some situations for which the re-randomisation design may be useful even if the assumption of constant treatment effect does not hold. For interventions which are intended to be used on a repeated basis (e.g. in situations where most patients require treatment a large number of times), the average treatment effect across randomisation periods may be more relevant to real world practice than the treatment effect from the first randomisation period (which would be obtained from a parallel group design), as this ‘average’ treatment effect is more reflective of what occurs on a per-episode basis.

For example, consider an intervention that is very effective the first time it is given, but becomes gradually less effective each subsequent time it is used. If this intervention will be used on a repeated basis for most patients in routine clinical practice, then the treatment effect from the first time it is given may be a misleading representation of what will occur for the majority of times it is used. Conversely, the average treatment effect across randomisation periods will not be a perfect representation of what occurs each time the intervention is used, but it is likely to be a better representation than one based on only the first randomisation period. Furthermore, utilising a re-randomisation design allows the estimation of the treatment effect in each separate randomisation period, allowing investigators to determine whether the treatment does indeed maintain its effectiveness when used on a recurring basis.

One issue to consider regarding the assumption of constant treatment effect is whether we should formally check this assumption during the analysis. For example, we could fit a treatment-by-randomisation period interaction to see whether there is evidence that the treatment effect varies over time. If there is evidence of an interaction, we could then use data from the first period only to estimate the treatment effect (providing a similar estimate to that of a parallel group trial). If there is insufficient evidence of an interaction, one could use data from all randomisation periods to estimate the treatment effect.

In practice, we would not recommend such an approach. This issue is similar to that of crossover trials (which also assumes the treatment effect is constant across periods) and factorial trials (which usually assumes there is no interaction between treatment arms). Research has shown that in both cases, choosing a method of analysis based upon the results of an interaction test will lead to biased estimates of treatment effect and an increased type I error rate [22, 23]. Therefore, we do not recommend formal assessment of the assumption of whether the treatment effect is constant across randomisation periods to inform the primary method of analysis; instead, we recommend only undertaking a re-randomisation design if (a) there is sufficient confidence that the assumption of constant treatment effect will hold; or (b) even if this assumption does not hold, the average treatment effect across randomisation periods is of interest.

### Covariate adjustment in re-randomisation trials

In many situations it may be useful to adjust for factors associated with the re-randomisation design, such as randomisation period, or the number of previous allocations to the intervention or the control. For example, if patient outcomes systematically differ across randomisation periods, adjustment for period in the analysis will increase power. If the effects of the intervention or control (or both) carry over into subsequent randomisation periods, adjustment for the number of previous allocations to each will ensure unbiased estimates of treatment effect when conducting an analysis which adjusts for patient-effects, and will increase power when conducting an unadjusted analysis. Because the method of analysis needs to be specified prior to seeing the data, we recommend routinely adjusting for these factors in the analysis of trials employing re-randomisation. This approach will ensure unbiased estimates and increased power in the above situations, and should lead to only a small loss in power when there are no differences across periods, and when treatment effects do not carry over (provided the number of randomisation periods is not too large).

In some cases, we may suspect that patients who are enrolled a large number of times are different than those enrolled a small number of times, and that adjustment for the total number of times enrolled will increase power. However, this is a post-randomisation factor, as it conditions on the future (i.e. whether the patient will require further treatment after the current randomisation period is finished). Including this in the analysis model will therefore lead to bias, and should be avoided.

### Re-randomisation with a small number of patients

Many of the results presented in this paper assume a large sample size. Our simulation study demonstrated that with a very small number of patients, some results may not hold, and there is a risk of an increased type I error rate. We found that adjustment for randomisation period in the analysis controlled the type I error rate, however given the limited number of simulation scenarios we ran, further research into the properties of the re-randomisation design with a small number of patients is necessary.

### Re-randomisation vs. crossover designs

Re-randomisation and crossover designs both allow patients to be included in the trial for multiple treatment periods, and therefore both could be used in certain settings. As such, it is worth exploring their differences to determine in which circumstances each design might be preferred. There are two key differences between the two designs: (a) the number of randomisation periods for each patient; and (b) the method of randomisation. We discuss each of these differences in turn.

In a crossover trial, the number of randomisation periods is the same for each patient, and is specified in advance. This design should therefore only be used when it is known in advance that each patient will require treatment at least the specified number of times during the trial period. Conversely, in a re-randomisation trial, each patient can have a different number of randomisation periods, and this number does not need to be specified in advance; it is based on the treatment requirements for each individual patient during the recruitment period. Therefore, the re-randomisation design can be used in a wider range of settings than the crossover design.

The second key difference between the two designs is the method of randomisation. Under the re-randomisation design, randomisations for the same patient are performed independently for each randomisation period. Patients may receive both the intervention and control, but are not ‘forced’ to do so by the randomisation procedure. Consequently, a within-patient comparison is only available for a subset of patients. Conversely, the classic ‘AB/BA’ crossover design does force patients to receive both the intervention and control. A within-patient comparison will then be available for all patients. As a result of the greater use of within-patient comparisons, crossover trials will have higher power than re-randomisation trials.

One disadvantage of forcing each patient to receive both the intervention and the control (as in a crossover trial) is that this approach can lead to bias if only a certain subset of patients return for their subsequent randomisation periods; this issue was discussed earlier in the methods section.

Another advantage of the re-randomisation design is that it can still provide unbiased estimates when the effects of the different treatment arms differentially carry over into subsequent randomisation periods. Conversely, most types of crossover designs (particularly those with two periods) cannot account for differential carryover, and will produce biased estimates in these scenarios (Balaam’s design is a notable exception [24]).

Therefore, if it is known in advance that each patient will require treatment a specified number of times during the trial period, there is little risk of patients dropping out prior to completing each treatment period, and treatment effects are unlikely to differentially carryover into subsequent periods, an AB/BA crossover design may be preferred as it will increase power compared to a re-randomisation design. However, if any of these conditions are in doubt, a re-randomisation design may be preferred, as it will reduce the risk of bias compared to a crossover design.

### Use of re-randomisation in open-label trials

In some trials, patients may have a strong preference for a particular treatment. For example, they may enrol in the trial hoping to receive the new intervention. This type of patient preference can have implications for open-label trials where patients are aware of their treatment allocation. If patients are allocated to their non-preferred treatment group, they may lose interest in the trial, and not adhere to treatment, or not return for their follow-up assessments.

Although this is a potential problem for any trial in which patients are aware of their treatment allocation, it may be exacerbated in trials using a re-randomisation design. For example, patients who experience a positive outcome during their first randomisation period may be upset if they do not receive the same treatment in subsequent randomisation periods. Conversely, patients who do not experience a positive outcome during their first randomisation period may re-enrol in the trial hoping to receive a different treatment in their second randomisation period; they may therefore be disappointed if they are allocated to the same treatment again. In both cases, patients may be less likely to adhere to treatment or to return for follow-up assessments during their subsequent randomisation periods, which could affect the validity of the trial results. Therefore, careful consideration regarding the possibility of a strong patient preference is required before using a re-randomisation design in trials for which patients are aware of their allocated treatment group.

### How many times can patients be re-randomised?

One issue to consider for the re-randomisation design is the number of times a patient can be re-randomised. Although in theory this could occur as often as treatment is required by the patient during the recruitment period, in practice in may be desirable to put an upper limit on this to avoid situations where a small number of patients contribute a large proportion of the total observations.

There are benefits and disadvantages to both lower and higher upper limits. Allowing a high number of re-randomisations will increase the recruitment rate, and will also increase power if the analysis adjusts for patient-effects. It may also increase generalizability by ensuring that patients who require treatment more often in usual clinical practice make up a representative proportion of the trial observations.

However, it could also reduce generalizability if a small number of patients make up the bulk of observations. For example, consider a trial where 20 patients contribute 200 observations; these patients may be less representative of the study population than 200 individual patients would be. A stricter upper limit, would negate this disadvantage, but would also reduce the recruitment rate. The choice of upper limit will likely depend on specific study characteristics, such as the overall sample size, expected recruitment rate, how often patients are expected to require treatment throughout the course of the study, and whether patients who require treatment much more frequently are likely to be systematically different to those who require treatment less frequently.

### When can a patient be re-randomised?

One of the requirements of the re-randomisation design is that the follow-up period from the previous enrolment must be completed before a patient can be re-randomised. However, it is possible to add a further requirement that patients must wait an additional period of time after completion of their previous follow-up period before they can be re-randomised (a washout period).

The primary benefit of a washout period is that it may increase the likelihood of the treatment effect being constant across all randomisations. This is particularly the case when there is a concern that the effectiveness of a treatment may vary according to which treatment the patient received in their previous randomisation period. This may be more likely to occur in drug trials, where the effects of the treatment received during the second randomisation period may be altered if some of the treatment received during the first randomisation period is still in the patient’s system. This is a common concern in crossover trials [23]. A washout period reduces the likelihood of the treatment effect being influenced by the treatment received in the previous period, as the effects of the treatment given in the previous period have had more time to wear off.

However, if the treatment effect is unlikely to be affected by the treatment received in the previous period, then allowing patients to be re-randomised as soon as the follow-up period from their previous randomisation period is complete, without need for a further washout period, has two advantages. The first is that it can increase the recruitment rate, as patients can be re-randomised more quickly. Second, it is more reflective of what happens in practice (i.e. patients receive treatment when required, regardless of how soon after completing the previous treatment period this is), and therefore will result in a more pragmatic design, which may lead to greater external validity [25].

### How should patients be consented for re-randomised trials?

In a re-randomisation design, patient consent could be taken in a number of ways. For example, they could be consented:

- At each presentation
- For a specified period of time (i.e. to be enrolled into the trial and randomised for any presentations that occur within the next 6 months)
- At their initial presentation, and then confirm if they wish to be included in the trial at each subsequent presentation.

Further research is needed on this issue to determine which approach is most acceptable to patients and trialists, and it may be that the ideal consent model depends on specific trial characteristics. In some cases, it may be preferable to offer the option of which consent model each patient would like to follow.

### Accidental re-randomisation

In some parallel group trials for which patients are only eligible to be enrolled once, some patients may accidentally be enrolled a second time [26]. This may occur if a new researcher is in charge of recruiting and enrolling patients and does not realise the patient has already been enrolled, or if the patient is enrolled at a different centre to their initial enrolment.

There are two options regarding the analysis in this situation: the first is to exclude the patient from the analysis, and the second is to include them. Excluding patients from the analysis is generally an unsatisfactory approach, as it goes against the intention-to-treat principle, and may lead to a loss in power due to a smaller sample size. Therefore, we do not recommend excluding patients who were accidentally re-randomised from the analysis. The one exception to this is if (a) the patient was re-enrolled before their follow-up period for the previous randomisation was complete; or (b) the treatment effect is unlikely to be the same across randomisation periods. In either of these scenarios, including these patients in the analysis could lead to biased estimates of treatment effect, and so excluding them may be a preferable option.

If patients who were accidentally re-randomised are included in the analysis, it is likely that an unadjusted analysis (ignoring ‘patient’ in the analysis) will be the preferred analysis strategy, as there are likely to be a small number of patients with multiple observations. In this situation, an adjusted analysis (including ‘patient’) is unlikely to increase power compared to an unadjusted analysis, and may be unstable due to the small number of clusters.

### Applying re-randomisation to the SWIM trial

We now discuss some of the considerations above in the context of applying a re-randomisation design to the SWIM trial.

#### Is the assumption of constant treatment effect reasonable?

The first and most important issue is whether it is reasonable to assume the treatment effect will be constant across randomisation periods. Ibuprofen is commonly used on a repeated basis, and there is no evidence that we are aware of to suggest that its effectiveness varies over time. In SWIM, ibuprofen was given for a maximum of four days; given the follow-up period is 4 weeks after hospital discharge, the ibuprofen should be gone from the patient’s system by the time they are re-randomised.

It is also worth noting that ibuprofen (if shown to be effective) would be given on a repeated basis to patients each time they presented with an acute sickle cell pain crisis. As such, even if the effectiveness of ibuprofen varies over time (e.g. it becomes more or less effective each time it is given), the average treatment effect over all randomisation periods is more relevant than the treatment effect the first time it is given (as would be obtained from a parallel group trial), as this more closely reflects clinical practice.

Therefore, we conclude that (a) the assumption that the treatment effect will be constant across randomisation periods seems likely; and (b) even if this assumption is incorrect, we are more interested in the average treatment effect across randomisation periods than the treatment effect in the first randomisation period. Therefore, the re-randomisation design is appropriate for SWIM.

#### Should we limit the number of times each patient can be enrolled?

The second issue to consider is the number of times each patient could be enrolled in the trial. One concern is that patients who present to hospital very frequently may be different than those who present less frequently. For example, they may have different levels of pain, or may respond differently to treatment. It may therefore be useful to limit the number of times each patient can be enrolled to ensure results are not dominated by the subset of patients who require treatment the most frequently. Although there is no right answer for the upper limit, choosing an upper limit in the range of 4–8 should increase the recruitment rate enough to make recruitment more feasible, while also ensuring no one patient unduly influences the results.

#### Should we introduce an extra washout period before patients can be re-enrolled?

The next issue to consider is *when* patients can be re-randomised, that is, whether they can be re-enrolled immediately after finishing their follow-up period, or whether it would be beneficial to require they wait an additional period of time before being re-enrolled. As above, there is no right answer to this question. The primary reason to introduce an extra washout period is to reduce the risk that the treatment effect is impacted based on the treatment given in the previous randomisation period. However, as discussed previously, ibuprofen should be out of the patient’s system by the end of their follow-up period, so it seems unlikely that the treatment effect in any randomisation period could be influenced by the treatment received in the previous period. Therefore, it seems reasonable to allow patients to be eligible for re-enrollment as soon as their follow-up period is complete.

#### How should we analyse SWIM?

In SWIM, we would expect a large proportion of patients to be enrolled on multiple occasions. Therefore, adjusting for patient-effects in the analysis is likely to lead to increased power compared to a parallel group trial. We would therefore use a mixed-effects linear regression model, with a random intercept for patient.

We would not expect outcomes to vary across randomisation periods, or for the intervention or control effects to carry over into subsequent randomisation periods. However, we would adjust for these covariates regardless, as there is likely to be little cost to doing so, and this approach will ensure unbiased estimates and increased power if we are wrong.

## Discussion

We have described a re-randomisation design, which allows patients who have completed their follow-up period to be re-randomised if they require further treatment. This design focuses on recruiting a specified number of observations, rather than patients. Importantly, the re-randomisation design does not require the number of times each patient is included to be specified in advance; instead, this depends on the specific needs of each individual patient, and so each patient can be enrolled a different number of times. This flexibility allows the re-randomisation design to be used in a wide variety of settings, including scenarios where the number of treatment episodes for each patient is not known in advance.

Because patients are allowed to contribute multiple observations, the re-randomisation design will require fewer overall patients than a parallel design; this could lead to lower costs, improved recruitment rate, and shorter trial durations. Additionally, when the correlation between observations from the same patient is accounted for in the analysis, this design will typically have higher power than a parallel group trial with an equivalent number of observations. This means that the re-randomisation design could both increase the recruitment rate while simultaneously reducing the overall number of observations required, compared with a parallel group trial. This approach could be particularly useful in trials of rare diseases, where recruitment can be particularly challenging.

## Conclusion

If used appropriately, the re-randomisation design can increase the recruitment rate for clinical trials while still providing an unbiased estimate of treatment effect and correct type I error rates. In many situations, it can increase the power compared to a parallel group design with an equivalent number of observations.

## Additional file

Additional file 1:
**A re-randomisation design for clinical trials - Online appendix.** (DOCX 61 kb)

## Abbreviations

GEE: Generalised estimating equations
RCT: Randomised controlled trial
