# Supplementary material for: A re-randomisation design for clinical trials
Source: BMC Med Res Methodol. 2015 Nov 5;15:96. doi: 10.1186/s12874-015-0082-2 (PMC4634916; doi:10.1186/s12874-015-0082-2)
Supplement: Additional file 1: — A re-randomisation design for clinical trials - Online appendix. (DOCX 61 kb) [file 12874_2015_82_MOESM1_ESM.docx]

**A re-randomisation design for clinical trials** **- Online appendix**

**Outline**

- **Section 1:** Estimated treatment effect from an unadjusted analysis
- **Section 2:** Equivalence in power between unadjusted analysis from a re-randomisation design and a parallel group trial with the same number of observations
- **Section 3:** Estimated treatment effect from adjusted analysis, and comparison of power between adjusted and unadjusted analyses
- **Section 4:** Potential effects of misspecification on bias of the estimated treatment effects
- **Section 5:** Simulation design and results for binary outcomes

**Section 1: Estimated treatment effect from an unadjusted analysis**

We evaluate the estimated treatment effect for an analysis which ignores patient effects (i.e. treats all observations as independent, even those from the same patient). For simplicity, we only consider the situation where patients are randomised up to a maximum of two times.

This analysis model for a continuous outcome follows the general form:

$$Y_{ij}=\alpha+\beta X_{ij}+\epsilon_{ij} (A1)$$

where $Y_{ij}$ is the outcome from the *j*th randomisation of the *i*th patient, $X_{ij}$ is a binary variable indicating whether patient *i* received treatment A or B during their *j*th randomisation (where A indicates the intervention and B the control), $\alpha$ is the mean outcome in patients who receive B, and $\beta$ is the treatment effect.

Let:

*n* = the number of patients ***initially*** randomised in each treatment group

*p* = proportion of patients in each treatment group who are re-randomised

Var(*Y_ij_*) = $\sigma^{2}$

Corr(*Y_i1_*, *Y_i2_*) = $\vartheta$

Note that the total number of observations in each group is: *n*(1+*p*).

Table 1 shows the six possible treatment sequences for this scenario, and the number of patients who experience each, and the expected value of the outcome for each period under model (A1). For simplicity, we assume that $\alpha=0$.

**Table 1: Possible treatment sequences under the re-randomisation design with a maximum of two randomisation periods**

|  | Treatment | | Expected values | | Number of allocations | |
| --- | --- | --- | --- | --- | --- | --- |
| Sequence | Period 1 | Period 2 | Period 1 | Period 2 | Period 1 | Period 2 |
| 1 | A |  | $\beta$ |  | n(1-p) |  |
| 2 | A | B | $\beta$ | $0$ | np/2 | np/2 |
| 3 | A | A | $\beta$ | $\beta$ | np/2 | np/2 |
| 4 | B |  | 0 |  | n(1-p) |  |
| 5 | B | A | 0 | $\beta$ | np/2 | np/2 |
| 6 | B | B | 0 | $0$ | np/2 | np/2 |

*Treatment A indicates the intervention, and treatment B indicates the control

The unadjusted estimator is

$${\bar{Y}_{A}-\bar{Y}}_{B}=\frac{\sum_{i,j\in A} Y_{ij}-\sum_{i,j\in B} Y_{ij}}{n\left( 1+p \right)}$$

The numerator can be expanded into its components from each sequence as

$$\sum_{i,j\in A} Y_{ij}-\sum_{i,j\in B} Y_{ij}=\sum_{i\in A} {(Y}_{i1})+\sum_{i\in AA} \left( Y_{i1}+Y_{i2} \right)+\sum_{i\in AB} \left( Y_{i1}-Y_{i2} \right)-\sum_{i\in B} (Y_{i1})-\sum_{i\in BB} \left( Y_{i1}+Y_{i2} \right)-\sum_{i\in BA} \left( Y_{i1}-Y_{i2} \right)$$

And for convenience we write this as $S_{A}+S_{AA}+S_{AB}-S_{B}-S_{BB}-S_{BA}$

We now write this in terms of three estimators of the treatment effect – two using between-subjects information and one using within-subjects information:

$$\text{Between-subjects A vs B:} \hat{\beta}_{B1}=\frac{S_{A}-S_{B}}{n\left( 1-p \right)}$$

$$\text{Between subjects AA vs BB:} \hat{\beta}_{B2}=\frac{S_{AA}-S_{BB}}{np} (A2)$$

$$\text{Within-subjects AB and BA:} \hat{\beta}_{W}=\frac{S_{AB}-S_{BA}}{np}$$

We can then write the unadjusted estimator as a weighted average of these three components:

$$\bar{Y}_{A}-\bar{Y}_{B}=\frac{1}{1+p}\left( \left( 1-p \right)\hat{\beta}_{B1}+p\hat{\beta}_{B2}+p\hat{\beta}_{W} \right) (A3)$$

Therefore the unadjusted estimator will be unbiased for the treatment effect $\beta$ when each of the three $\hat{\beta}_{j}, j\in\{B1, B2, W\}$ are unbiased, which is the case under model (A1) as is shown in section 4.

**Section 2: Equivalence in power between unadjusted analysis from a re-randomisation design and a parallel group trial with the same number of observations**

Under certain conditions, the power from a re-randomisation design using an unadjusted analysis (model (A1)) will be equivalent to that of a parallel group trial with the same number of observations.

We consider the situation where a subset of patients are re-randomised, and for simplicity, focus on scenarios where patients are randomised a maximum of two times.

*Y_ij_* is as defined as earlier, with *i* representing patients, and *j* representing randomisation periods (with *j*=1,2).

As above, let:

*n* = the number of patients ***initially*** randomised in each treatment group

*p* = proportion of patients in each treatment group who are re-randomised

Var(*Y_ij_*) = $\sigma^{2}$

Corr(*Y_i1_*, *Y_i2_*) = $\vartheta$

Note that the total number of observations in each group is: *n*(1+*p*).

The variance of the treatment effect from a parallel group trial with *n*(1+*p*) patients in each treatment arm is:

$$\text{Var}_{pg}= \frac{2\sigma^{2}}{n(1+p)}$$

We now demonstrate that the variance of the treatment effect from a parallel group trial analysed using model (A1) above (i.e. assuming that all observations are independent) is equivalent to $V_{pg}$. This requires that the variance $\sigma^{2}$ is constant across each randomisation period, and that there are no systematic differences between (a) patients who are re-randomised vs. those who are not; and (b) randomisation periods.

To obtain the variance of the unadjusted estimator, note that for treatment sequences AA and BB: $V\left( Y_{i1}+Y_{i2} \right)=2\sigma^{2}(1+\vartheta)$, and for treatment sequences AB and BA: $V\left( Y_{i1}-Y_{i2} \right)=2\sigma^{2}(1-\vartheta)$.

Then

$$\mathrm{Var}\left( \hat{\beta}_{B1} \right)=\frac{2\sigma^{2}}{n\left( 1-p \right)}$$

$$\mathrm{Var}\left( \hat{\beta}_{B2} \right)=\frac{2\sigma^{2}\left( 1+\vartheta\right)}{np} (A4)$$

$$\mathrm{Var}\left( \hat{\beta}_{W} \right)=\frac{2\sigma^{2}\left( 1-\vartheta\right)}{np}$$

Therefore

$$\mathrm{Var}\left( \bar{Y}_{A}-\bar{Y}_{B} \right)=\frac{1}{\left( 1+p \right)^{2}}\left[ \left( 1-p \right)^{2}\frac{2\sigma^{2}}{n\left( 1-p \right)}+p^{2}\frac{2\sigma^{2}\left( 1+\vartheta\right)}{np}+p^{2}\frac{2\sigma^{2}\left( 1-\vartheta\right)}{np} \right]$$

$$=\frac{2\sigma^{2}}{n\left( 1+p \right)}$$

Therefore, the variance for the unadjusted treatment effect under a re-randomisation design (which does not account for patient effects in the analysis) is the same as the variance under a parallel group design with an equivalent number of observations. It follows from this that the power for the two designs will be equivalent.

It also demonstrates that one can ignore the within-subject correlation in the analysis using the unadjusted estimator and obtain valid standard errors.

**Section 3: Estimated treatment effect from adjusted analysis, and comparison of power between adjusted and unadjusted analyses**

A mixed-effects model with a continuous outcome takes the general form:

$$Y_{ij}=\alpha+\beta X_{ij}+u_{i}+\varepsilon_{ij}$$

where $u_{i}$ is a random-effect for the *i*th patient, and is generally assumed to follow a normal distribution with mean 0.

Under a mixed model with random subject effects the treatment effect estimator is a weighted average of between and within-subject effects, with weights equal to the reciprocal of the variances of the three components in (A2), whose variances are provided in (A4).:

$$\hat{\beta}_{adj}=\sum_{j} {w_{j}\hat{\beta}}_{j}/\sum_{j} w_{j}$$

$$\text{where} w_{j}=\frac{1}{\text{Var}\left( \hat{\beta}_{j} \right)}, j\in\left\{ B1, B2, W \right\}$$

and

$$\text{Var}\left( \hat{\beta}_{adj} \right)=\frac{1}{\sum_{j} w_{j}}$$

After some routine algebra one obtains

$$\text{Var}\left( \hat{\beta}_{adj} \right)=\frac{2\sigma^{2}}{n}\frac{1-\vartheta^{2}}{1+p-\left( 1-p \right)\vartheta^{2}}$$

A little further algebra then shows

$$\text{Var}\left( \hat{\beta}_{adj} \right)\leq\text{Var}\left( \bar{Y}_{A}-\bar{Y}_{B} \right)=\frac{2\sigma^{2}}{n\left( 1+p \right)}$$

with equality only when $p=0$ or $\vartheta=0$, which occurs when either there is no re-randomisation, or when repeated observations of an individual are uncorrelated.

Therefore accounting for subject effects under model (A1) with a mixed model (or GEE with exchangeable correlation) will produce a smaller variance than using an unadjusted analysis. Equivalently, this will lead to increase power.

**Section 4: Potential effects of misspecification on bias of the estimated treatment effects**

We now evaluate the bias in the estimated treatment effects of the adjusted and unadjusted estimates from a re-randomisation design. We consider seven scenarios:

- **Scenario 0**: Model (A1) is correct
- **Scenario 1**: Patients who are re-randomised are sicker than those who are not
- **Scenario 2**: Patients who experienced a poor outcome during their first randomisation are more likely to be re-randomised
- **Scenario 3**: Patients who received the intervention during their first randomisation are more likely to be re-randomised
- **Scenario 4:** Patients who received the control during their first randomisation are more likely to be re-randomised
- **Scenario 5:** Patients’ health status changes for their subsequent re-randomisation in both treatment groups
- **Scenario 6:** patients’ health status changes for their subsequent re-randomisation if they were allocated to the intervention group for their current randomisation.

***Scenario 0: Model (A1) is correct***

Assume *n* subjects initially randomised to A and *n* randomised to B. A proportion *p* of subjects initially randomised to A are re-randomised, and proportion *p* of patients initially randomised to B are re-randomised. For simplicity (and without loss of generality) we assume that $\alpha=0$. Table 2 shows the expected outcomes for patients under this scenario.

**Table 2: Expected outcomes for patients under scenario 0**

|  | Treatment | | Expected values | | Number of allocations | |
| --- | --- | --- | --- | --- | --- | --- |
| Sequence | Period 1 | Period 2 | Period 1 | Period 2 | Period 1 | Period 2 |
| 1 | A |  | $\beta$ |  | n(1-p) |  |
| 2 | A | B | $\beta$ | 0 | np/2 | np/2 |
| 3 | A | A | $\beta$ | $\beta$ | np/2 | np/2 |
| 4 | B |  | $0$ |  | n(1-p) |  |
| 5 | B | A | $0$ | $\beta$ | np/2 | np/2 |
| 6 | B | B | 0 |  | np/2 | np/2 |

From Table 2, we see that:

$$\text{E}\left( \hat{\beta}_{B1} \right)=\frac{n\left( 1-p \right)\beta-n\left( 1-p \right)0}{n\left( 1-p \right)}=\beta$$

$$\text{E}\left( \hat{\beta}_{B2} \right)=\frac{\frac{np}{2}\beta+\frac{np}{2}\beta-\frac{np}{2}0-\frac{np}{2}0}{np}=\beta$$

$$\text{E}\left( \hat{\beta}_{W} \right)=\frac{\frac{np}{2}\beta-\frac{np}{2}0-\left( \frac{np}{2}0-\frac{np}{2}\beta\right)}{np}=\beta$$

And therefore from (A3)

$${\text{E}(\bar{Y}}_{A}-\bar{Y}_{B}) =\frac{1}{1+p}\left( \left( 1-p \right){\text{E}(\hat{\beta}}_{B1})+p\text{E}(\hat{\beta}_{B2})+p{\text{E}(\hat{\beta}}_{W}) \right)=\beta$$

Similarly,

$${\text{E}(\hat{\beta}}_{adj})=\sum_{j} {w_{j}\text{E}(\hat{\beta}}_{j})/\sum_{j} w_{j} =\beta$$

where $w_{j}$ are defined in (A4).

***Scenario 1: Patients who are re-randomised are sicker than those who are not***

The correct model here is defined as:

$$Y_{ij}=\alpha+\beta X_{ij}+\theta Z_{ij}+u_{i}+\varepsilon_{ij}$$

where $Z_{ij}$is a binary variable indicating whether the patient requires re-randomisation or not, and $\theta$ indicates the average difference in outcomes between patients who were re-randomised compared to those who were not.

For simplicity (and without loss of generality) we assume that $\alpha=0$. Table 3 shows the expected outcomes for patients under this scenario.

**Table 3: Expected outcomes for patients under scenario 1**

|  | Treatment | | Expected values | | Number of allocations | |
| --- | --- | --- | --- | --- | --- | --- |
| Sequence | Period 1 | Period 2 | Period 1 | Period 2 | Period 1 | Period 2 |
| 1 | A |  | $\beta$ |  | n(1-p) |  |
| 2 | A | B | $\beta+\theta$ | $\theta$ | np/2 | np/2 |
| 3 | A | A | $\beta+\theta$ | $\beta+\theta$ | np/2 | np/2 |
| 4 | B |  | 0 |  | n(1-p) |  |
| 5 | B | A | $\theta$ | $\beta+\theta$ | np/2 | np/2 |
| 6 | B | B | $\theta$ | $\theta$ | np/2 | np/2 |

Therefore:

$$\text{E}\left( \hat{\beta}_{B1} \right)=\frac{n\left( 1-p \right)\beta-n\left( 1-p \right)0}{n\left( 1-p \right)}=\beta$$

$$\text{E}\left( \hat{\beta}_{B2} \right)=\frac{\frac{np}{2}\left( \beta+\theta\right)+\frac{np}{2}(\beta+\theta)-\frac{np}{2}\theta-\frac{np}{2}\theta}{np}=\beta$$

$$\text{E}\left( \hat{\beta}_{W} \right)=\frac{\frac{np}{2}(\beta+\theta)-\frac{np}{2}\theta-\left( \frac{np}{2}\theta-\frac{np}{2}(\beta+\theta) \right)}{np}=\beta$$

And therefore both the unadjusted and adjusted estimators are unbiased.

***Scenario 2: Patients who experienced a poor outcome during their first randomisation are more likely to be re-randomised***

Patients with initial observation *Y*<*c* are re-randomised for some constant *c*.

Therefore the probability of re-randomisation is $\kappa_{A}=P \left( Y<C | \text{Trt A} \right), \kappa_{B}=P \left( Y<C | \text{Trt B} \right),$

However the outcome from the second allocation is not influenced by the first, apart from the correlation $\vartheta$, so the expected outcomes in each cell are the same as in Scenario 0. This is shown in table 4.

**Table 4: Expected outcomes for patients under scenario 2**

|  | Treatment | | Expected values | | Number of allocations | |
| --- | --- | --- | --- | --- | --- | --- |
| Sequence | Period 1 | Period 2 | Period 1 | Period 2 | Period 1 | Period 2 |
| 1 | A |  | $\beta$ |  | n(1-$\kappa$_A_) |  |
| 2 | A | B | $\beta$ | $0$ | n $\kappa$_A_ /2 | n $\kappa$_A_ /2 |
| 3 | A | A | $\beta$ | $\beta$ | n $\kappa$_A_ /2 | n $\kappa$_A_ /2 |
| 4 | B |  | 0 |  | n(1-$\kappa$_B_) |  |
| 5 | B | A | 0 | $\beta$ | n $\kappa$_B_ /2 | n $\kappa$_B_ /2 |
| 6 | B | B | 0 | $0$ | n $\kappa$_B_ /2 | n $\kappa$_A_ /2 |

Here we cannot partition ${\bar{Y}_{A}-\bar{Y}}_{B}$ nicely into the three components because $\kappa_{A}\neq\kappa_{B}$ so we use its full expression:

$${\bar{Y}_{A}-\bar{Y}}_{B}$$

$$=\frac{\sum_{i\in A} {(Y}_{i1})+\sum_{i\in AA} \left( Y_{i1}+Y_{i2} \right)+\sum_{i\in AB} \left( Y_{i1}-Y_{i2} \right)-\sum_{i\in B} \left( Y_{i1} \right)-\sum_{i\in BB} \left( Y_{i1}+Y_{i2} \right)-\sum_{i\in BA} \left( Y_{i1}-Y_{i2} \right)}{n\left( 1+\frac{\kappa_{A}+\kappa_{B}}{2} \right)}$$

And hence

$$E\left( \bar{Y}_{A}-\bar{Y}_{B} \right)=\frac{\left( 1-\kappa_{A} \right)\beta+\frac{\kappa_{A}}{2}\left( \beta+\beta\right)+\frac{\kappa_{A}}{2}\left( \beta-0 \right)-\left( 1-\kappa_{B} \right)0-\frac{\kappa_{B}}{2}\left( 0+0 \right)-\frac{\kappa_{B}}{2}\left( 0-\beta\right)}{1+\frac{\kappa_{A}+\kappa_{B}}{2}}$$

$$=\beta$$

For the adjusted estimator, we extend the previous definitions to unequal $p_{A},p_{B}$ and replacing with $\kappa_{A},\kappa_{B}$ as

$$\hat{\beta}_{B1}=\frac{\sum_{A} Y_{i1}}{n(1-\kappa_{A})}-\frac{\sum_{B} Y_{i1}}{n(1-\kappa_{B})}$$

$$\hat{\beta}_{B2}=\frac{1}{2}\left( \frac{{\sum_{AA} Y}_{i1}+Y_{i2}}{\frac{n\kappa_{A}}{2}}-\frac{{\sum_{BB} Y}_{i1}+Y_{i2}}{\frac{n\kappa_{B}}{2}} \right)$$

$$\hat{\beta}_{W}=\frac{1}{2}\left( \frac{{\sum_{AB} Y}_{i1}-Y_{i2}}{\frac{n\kappa_{A}}{2}}-\frac{{\sum_{BA} Y}_{i1}-Y_{i2}}{\frac{n\kappa_{B}}{2}} \right)$$

Then, replacing $p$ by $\kappa$,

$$\text{E}\left( \hat{\beta}_{B1} \right)=\beta-0=\beta$$

$$\text{E}(\hat{\beta}_{B2})=\frac{1}{2}\left( \beta+\beta-\left( 0+0 \right) \right)=\beta$$

$$\text{E}\left( \hat{\beta}_{W} \right)=\frac{1}{2}\left( \beta-0-\left( 0-\beta\right) \right)=\beta$$

and therefore the adjusted estimator will also be unbiased.

***Scenarios 3 and 4: Patients who received the intervention (or control) during their first randomisation are more likely to be re-randomised***

Intervention patients are re-randomised with probability $p_{A}$ and control patients re-randomised with probability $p_{B}$.

The numerical simulations use $\left( p_{A},p_{B} \right)=(1,0)$ for Scenario 3 and $\left( p_{A},p_{B} \right)=(0,1)$ for Scenario 4.

Unbiasedness of the unadjusted and adjusted estimators follow directly from the arguments in Scenario 2 by replacing $\kappa_{A}, \kappa_{B}$ with $p_{A},p_{B}$.

***Scenario 5: Patients’ health status changes for their subsequent re-randomisation in both treatment groups***

The true model is:

$Y_{ij}=\alpha+\beta X_{ij}+\pi Z_{ij}+u_{i}+\varepsilon_{ij}$

where $Z_{ij}$is a categorical variable indicating the re-randomisation period, and $\pi$ represents the change in outcome for that re-randomisation period. We assume that $p_{A}$=$p_{B}$=$p$, and $\alpha=0.$ Table 5 shows the expected outcomes for patients under this scenario.

**Table 5: Expected outcomes for patients under scenario 5**

|  | Treatment | | Expected values | | Number subjects | |
| --- | --- | --- | --- | --- | --- | --- |
| Sequence | Period 1 | Period 2 | Period 1 | Period 2 | Period 1 | Period 2 |
| 1 | A |  | $\beta$ |  | n(1-p) |  |
| 2 | A | B | $\beta$ | $\pi$ | np/2 | np/2 |
| 3 | A | A | $\beta$ | $\beta$+$\pi$ | np/2 | np/2 |
| 4 | B |  | 0 |  | n(1-p) |  |
| 5 | B | A | 0 | $\beta+\pi$ | np/2 | np/2 |
| 6 | B | B | 0 | $\pi$ | np/2 | np/2 |

Therefore:

$$E\left( \hat{\beta}_{B1} \right)=\frac{n\left( 1-p \right)\beta-n\left( 1-p \right)0}{n\left( 1-p \right)}=\beta$$

$$E\left( \hat{\beta}_{B2} \right)=\frac{\frac{np}{2}\beta+\frac{np}{2}(\beta+\pi)-\frac{np}{2}0-\frac{np}{2}\pi}{np}=\beta$$

$$E\left( \hat{\beta}_{W} \right)=\frac{\frac{np}{2}\beta-\frac{np}{2}\pi-\left( \frac{np}{2}0-\frac{np}{2}(\beta+\pi) \right)}{np}=\beta$$

And therefore both the unadjusted and adjusted estimators are unbiased.

***Scenario 6: patients’ health status changes for their subsequent re-randomisation if they were allocated to the intervention group for their current randomisation.***

The true model is:

$$Y_{ij}=\alpha+\beta X_{ij}+\theta Z_{ij}+u_{i}+\varepsilon_{ij}$$

where $Z_{ij}$is a categorical variable indicating the number of times the patient has been allocated to the intervention in their previous randomisations, and $\theta$ is the effect on outcome for each additional previous time allocated to the intervention.

We assume that $p_{A}$=$p_{B}$=$p$, and $\alpha=0.$ Table 6 shows the expected outcomes for patients under this scenario.

**Table 6: Expected outcomes for patients under scenario 6**

|  | Treatment | | Expected values | | Number subjects | |
| --- | --- | --- | --- | --- | --- | --- |
| Sequence | Period 1 | Period 2 | Period 1 | Period 2 | Period 1 | Period 2 |
| 1 | A |  | $\beta$ |  | n(1-p) |  |
| 2 | A | B | $\beta$ | $\theta$ | np/2 | np/2 |
| 3 | A | A | $\beta$ | $\beta$+$\theta$ | np/2 | np/2 |
| 4 | B |  | 0 |  | n(1-p) |  |
| 5 | B | A | 0 | $\beta$ | np/2 | np/2 |
| 6 | B | B | 0 | $0$ | np/2 | np/2 |

Therefore:

$$E\left( \hat{\beta}_{B1} \right)=\frac{n\left( 1-p \right)\beta-n\left( 1-p \right)0}{n\left( 1-p \right)}=\beta$$

$$E\left( \hat{\beta}_{B2} \right)=\frac{\frac{np}{2}\beta+\frac{np}{2}(\beta+\theta)-\frac{np}{2}0-\frac{np}{2}0}{np}=\beta+\frac{\theta}{2}$$

$$E\left( \hat{\beta}_{W} \right)=\frac{\frac{np}{2}\beta-\frac{np}{2}\theta-\left( \frac{np}{2}0-\frac{np}{2}\beta\right)}{np}=\beta-\frac{\theta}{2}$$

Here the between-subjects estimator based on comparing A vs B is unbiased, but the between subject estimator comparing AA vs BB is biased, as is the within-subjects estimator.

However, recall from (A3) that the unadjusted estimator is a weighted average of these three estimators, and hence:

$${E(\bar{Y}}_{A}-\bar{Y}_{B})=\frac{1}{1+p}\left( \left( 1-p \right){E(\hat{\beta}}_{B1})+p{E(\hat{\beta}}_{B2})+p{E(\hat{\beta}}_{W}) \right)$$

$$=\frac{1}{1+p}\left( \left( 1-p \right)\beta+p\left( \beta+\frac{\theta}{2} \right)+p\left( \beta-\frac{\theta}{2} \right) \right)$$

$$=\beta$$

And therefore the unadjusted estimator is unbiased. Notice the between-subjects estimator $\hat{\beta}_{B2}$ and the within subjects estimator $\hat{\beta}_{W}$ exhibit bias of equal magnitude but in opposite directions, and receive the same weight $/(1+p)$ . As a result, their biases cancel perfectly.

For the adjusted estimator (which weights the $\hat{\beta}^{'}s$ by their inverse variances, $w_{j}$ defined in (A4))

$${\text{E}(\hat{\beta}}_{adj})=\sum_{j} {w_{j}\text{E}(\hat{\beta}}_{j})/\sum_{j} w_{j}$$

And after some routine but tedious algebra,

$$\text{E}\left( \hat{\beta}_{adj} \right)=\beta-\frac{p\vartheta^{*}}{1-\vartheta^{*2}+\vartheta^{*2}p+p}\theta$$

where $\vartheta^{*}$ is the probability limit of the sample ICC estimator under the misspecified model (A1). This estimator is obtained from the estimators of the variance components $\sigma_{u}^{2}$ and $\sigma_{\epsilon}^{2}$ of $u$ and $\epsilon$ in the random effects model. These estimated variance components are likely to be biased in the presence of misspecification of the model for the mean of Y.

Therefore the adjusted estimator is biased unless $\theta=0, or$ $p=0$ (no re-randomisation) or $\vartheta^{*}=0$ (which we conjecture will occur when $\vartheta=0$, i.e. no true correlation between repeated observations of individuals.).

**Section 5: Simulation design and results for binary outcomes**

We generated binary outcomes from the following model:

$$Y_{ij}^{*}=\alpha+\beta X_{ij}+u_{i}+\varepsilon_{ij}$$

where $Y_{ij}^{*}$is a latent outcome for the *j*th observation from the *i*th patient and the $u_{i}$ and $\varepsilon_{ij}$are normally distributed. Binary responses were generated as $Y_{ij}$= 1 if $Y_{ij}^{*}$> 0, and 0 otherwise. For all simulation scenarios we fixed the total variance as $\sigma_{u}^{2}+\sigma_{\varepsilon}^{2}=1$. This implies the data were generated from a Probit model. We allocated patients to one of two treatment arms using simple randomisation.

***Varying the ICC***

We varied the ICC on the latent outcome scale between 0.10, 0.25, 0.50, 0.75, and 0.90, while keeping the total variance ($\sigma_{u}^{2}+\sigma_{\varepsilon}^{2}$) fixed at 1 (i.e. for increasing ICCs, we increased $\sigma_{u}^{2}$ while reducing $\sigma_{\varepsilon}^{2}$). For each scenario, we kept the proportion of re-randomisations fixed. We used 200 observations (100 patients randomised once, 50 patients randomised twice). For each ICC value we also evaluated a set of completely independent observations (200 patients randomised once) to compare the re-randomisation and parallel group designs. It should be noted that the ICCs for the dichotomised binary outcomes will be less than the ICCs we specified on the latent scale, and as a result, the increase in power from an adjusted analysis will be less than the latent scale ICC would indicate.

***Varying the proportion of re-randomisations***

We varied the proportion of randomisations as follows: (a) 100 patients randomised once, 50 patients randomised twice; (b) 100 patients randomised once, 25 patients randomised four times; (c) 100 patients randomised twice; (d) 50 patients randomised four times; and (e) 25 patients randomised eight times. For each scenario we set the ICC to 0.50.

**Results**

Results are shown in figures 1 and 2. Results are similar to those seen with continuous outcomes. The gains in power from an adjusted analysis was less than in the continuous case, although this is likely a direct effect from having specified the ICCs on the latent scale rather than on the binary scale.

**Figure 1: Simulation results for binary outcomes across different ICC values**

We compared three methods of analysis: (a) analysis of a parallel group trial with 200 independent patients; (b) an unadjusted analysis (ignoring patient effects) of a re-randomisation design, with 100 patients randomised once, and 50 patients randomised twice; and (c) an adjusted analysis (accounting for patient effects using a mixed-effects model) of a re-randomisation design, with 100 patients randomised once, and 50 patients randomised twice. The treatment effect estimates from all three methods of analysis were unbiased. ICCs are specified on the latent outcome scale.

**Figure 2: Simulation results for binary outcomes across different re-randomisation proportions**

We compared two methods of analysis: (a) an unadjusted analysis (ignoring patient effects); and (b) an adjusted analysis (accounting for patient effects using a mixed-effects model). The ICC was set to 0.50 on the latent outcome scale for all scenarios. The estimated treatment effect was unbiased for both methods of analysis.
